# Relative influence of environmental factors on the timing and occurrence of multi-species coral reef fish aggregations

**DOI:** 10.1371/journal.pone.0209234

**Published:** 2018-12-21

**Authors:** Eric E. Fisher, John H. Choat, Mark I. McCormick, Mike Cappo

**Affiliations:** 1 College of Science and Engineering, James Cook University, Townville, Queensland, Australia; 2 Reef Education/ Reef Magic Cruises, Cairns, Queensland, Australia; 3 Australian Institute of Marine Science, Cape Cleveland, Queensland, Australia; Department of Agriculture and Water Resources, AUSTRALIA

## Abstract

Reef configuration and hydrodynamics were identified as the principle physical drivers behind coral reef fish aggregations on a mid-shelf patch reef in the northern section of the Great Barrier Reef (-16.845°, 146.23°). The study was carried out over a six-year period at a large reef pass on the oceanic margin of the northern Great Barrier Reef. Over this period (February 2006 –December 2012) tidal state, moon phase and surface seawater temperature were monitored. The timing of sampling was organised to assess variation in physical environment at daily, monthly, seasonal and annual time scales. Over these time scales, temporal patterns of occurrence of 10 species of coral reef fish from 5 families representing 5 defined trophic groups were monitored. The study incorporated 1,357 underwater visual census counts involving 402,370 fish and these estimates were collated with data on tidal state, water temperature, lunar and seasonal periodicity. Aggregated boosted regression trees analysed the univariate responses of fish abundance and species richness to the variation in the physical environment of the reef pass. Flood tides or when water flows from open water through the pass and into the Moore Reef lagoon had 2.3 times as many fish and 1.75 times as many species compared to counts made on ebb tides. Fish abundance was highest in late winter and spring months (Austral calendar), but notably when water temperatures were below the long-term mean of 27°C. Multivariate regression trees and Dufrêne-Legendre indicator predicted 4 out of 10 times the occurrence of all 10 species at any temporal scale ranging from hours to years. Flood tides were the principle driver underlying the occurrence of all 10 species regardless of their trophic classification and produced distinct seasonal assemblages, indicative of fishes aggregating to forage and reproduce.

## Introduction

A typical feature of reef-fish behaviour is the formation of aggregations for the purposes of reproduction and feeding at characteristic sites in coral reef systems. These behaviours are frequently observed in reef passes, which provide abrupt transitions between productive shallow benthic reef habitats and open ocean ecosystems [1–5]. Although tropical open oceanic environments are regarded as largely oligotrophic, they support large populations of macroplanktonic organisms including gelatinous zooplankters which make a substantial contribution to the flux of carbon and remineralization processes on adjacent reefs [6]. Oceanic waters adjacent to reefs are enhanced by hydrodynamic processes that induce upwelling of nutrient rich water [7–10]. In addition to the transport and redistribution of potential food items for planktonic and nektonic feeding reef fishes, reef passes are also associated with current systems that may redistribute eggs and developing larvae of spawning reef fishes [1, 11–13]. To date, much of the interest in reef fish-aggregations has focused on the significance of reproductive events and the role of the physical environment in determining the timing and intensity of spawning [14–16]. Recent studies have noted the critical importance of environmental influences, especially temperature, on initiating reproductive activities and the need to monitor variation in the physical environment at locations that support aggregative behaviours [17].

There is an extensive literature on the biological and environmental factors driving the formation of spawning aggregations [18]. Also, the formation of aggregations to exploit food items that are concentrated in reef passes contemporaneously with spawning aggregations has been described [5, 19, 20]. However, foraging aggregations may develop from the environment created by the interaction of reef geomorphology and hydrodynamic forces, that concentrate plankton, nekton and nutrients in reef passes [8, 12, 21]. The importance of environmental variables in determining the timing of aggregations has been noted in recent studies, such as current direction, time of day [16] and temperature [17], with respect to spawning events. However, given the complexity of reef pass environments an understanding of the triggers for the formation of fish aggregations requires a more comprehensive knowledge of the temporal pattern of environmental variation in reef passes. Hydrodynamic processes operate on a variety of timescales. These range from daily tidal cycles to monthly lunar cycles in the strength of tidal flows, seasonal trends where the environment is modified by changing temperatures and prevailing winds, through to inter-annual variation reflecting episodic trends including El Niño-Southern-Oscillation (ENSO) events. For this reason, long-term environmental monitoring at aggregation sites is critical. Moreover, such data sets are necessary for modelling responses in fish aggregation dynamics to climate change [17].

The present study adapts a widely accepted definition for fish spawning aggregations [22] and defines fish aggregations as: a repeatable gathering of conspecific fish at a predictable time and space with a four-fold increase in density compared with non-aggregating times. However, in nature many fish aggregations sites are used by many species, and such aggregations include a variety of trophic groups and interactions. These include egg predators exploiting the products of spawning aggregations [1, 19, 23], and large aggregations of reef fish that represent prey for larger predators which also congregate at such sites [4, 5, 24]. Finally, through defecation, reef-fish aggregations can subsidise nutrient inputs to reef ecosystems [25, 26] or directly augment food resources [27]. Clearly, fishes can form aggregations for a number of reasons that are not necessarily mutually exclusive, including mate selection, spawning, and foraging.

The goal of the present study was to examine the extent to which key features of the hydrodynamic environment predicted the occurrence of 10 fish species from five distinct trophic groups at a fish aggregation site over a six-year period. The hydrodynamic features chosen for this study ranged in temporal scale from daily (tidal regimes) too seasonal (water temperature) through to inter-annual periodicities. Using multivariate hierarchical analysis, we examined the temporal changes in fish assemblages at a fish aggregation site and behavioural observations over time enabled hypotheses to be advanced regarding the drivers for aggregations at particular times. We predicted from research at other aggregation sites that: a) aggregation of fishes for the purpose of reproduction would have a seasonal signature, synchronised by lunar and tidal cues to take advantage of strong currents that advect gametes away from reef heterotrophs [1, 19, 28]; b) tidal inputs of planktonic and smaller nekton may result in a consistent presence of planktivores and piscivorous fish predators [29, 30] and c) on short time scales reversals of the daily tidal cycle would be the most potent source of variation, with planktivores and their predators exploiting the incoming tidal flow, and reproductive aggregations and egg predators associated with the outgoing flow [11, 31]. Because of the requirement of intensive sampling at limited locations, long-term studies involve a trade-off between temporal and spatial monitoring. While marine macroecology has benefitted from the analysis of spatially extensive data sites, inferences about processes are best evaluated by dynamic data collected over important temporal scales [32] and the latter was the focus for the current study. The long-term monitoring of this single location yielded a unique and detailed perspective of the significant temporal drivers of fish abundance at a tropical multi-species fish aggregation site at the scales of years, seasons, lunar phases and tides.

## Methods

### Study site

**Fig 1:**
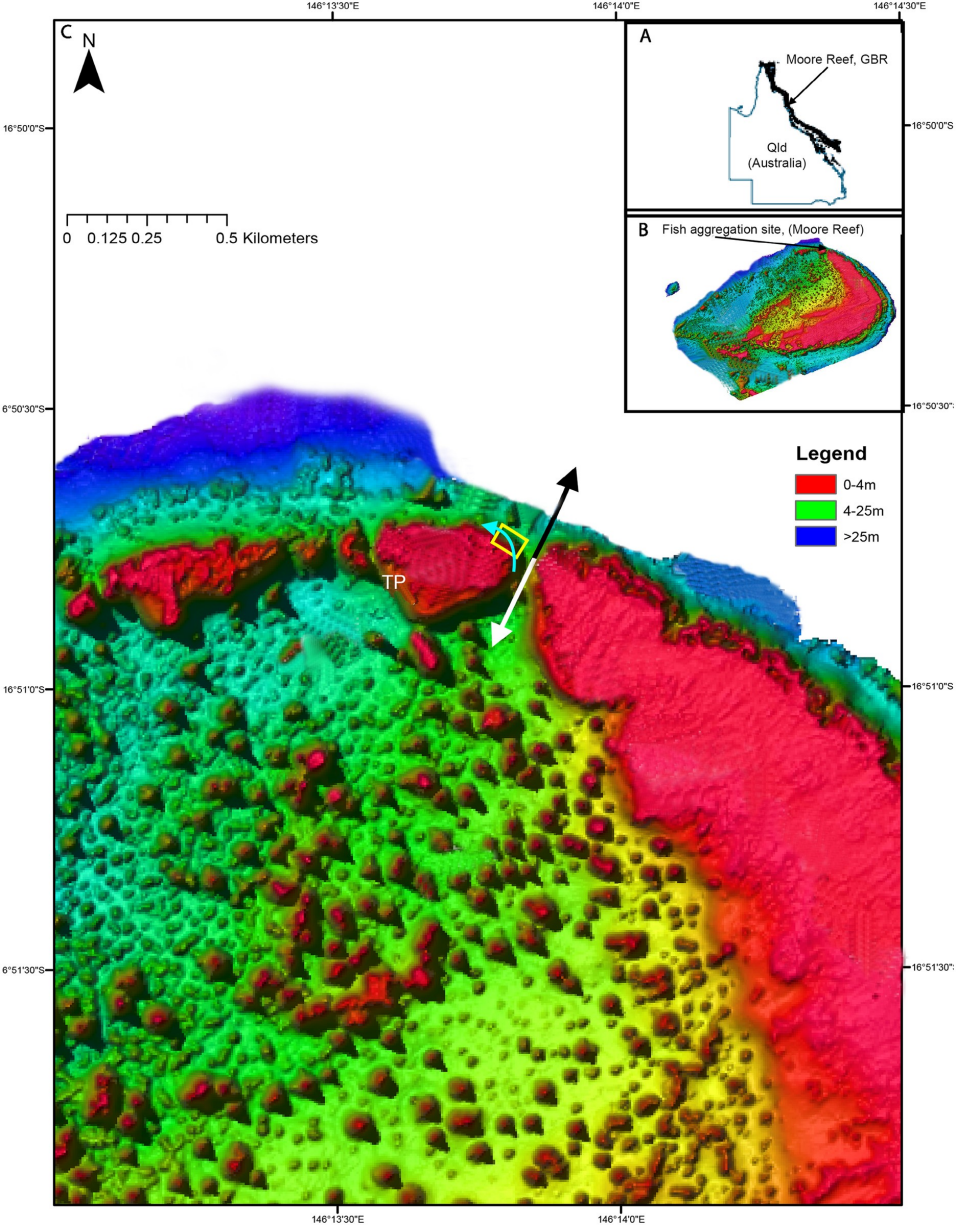
Site map of Moore Reef fish aggregation site. (A) The location of Moore Reef in the Great Barrier Reef and (B) the location of the fish aggregation site at Moore Reef. (C) Bathymetric map of reef scape at the Moore Reef fish aggregation site. The direction of flows through the passage are detailed by the white (flooding) and black (ebbing) arrows. The letters (TP) refer to the location of a commercial tourism platform and the underwater visual census path is represented by the light blue arrow passing through the fish aggregation area (yellow box). The lidar (light imaging, detection and ranging) bathymetry data of Moore Reef was courtesy of Dr Robin Beaman, James Cook University.

The study site (-16.845°, 146.23°), was situated approximately 55 km east of Cairns on the north-western side of Moore Reef, a mid-shelf patch reef located in the northern section of the Great Barrier Reef (GBR), Australia (Fig 1A). Moore Reef has a crescent shape, characterised by an extensive reef flat on the windward margins, with a well-developed reef front that semi-encloses a lagoon dominated by isolated patch reefs (Fig 1B). Several reef passages dissect the reef flat at the north-western corner of Moore Reef (Fig 1C). The fish aggregation site (FA) was in close proximity to a commercial tourism site that operates off a platform on the lagoon side of Moore Reef (~ 350 m direct line; Fig 1C), which facilitated the means to perform underwater visual census counts (UVC) at the FA regularly. The Great Barrier Reef Marine Park Authority (GBRMPA) have classed Moore Reef as Marine National Park Zone. The commercial tourism company operates under GBRMPA permit G10/33331.1. The research carried out in this study was non-evasive, but was conducted under GBRMPA research permit G12/35200.1. The UVC counts were conducted from February 2006 to November 2012. The larger reef passage is ~330 m in length, 60 to 250 m in width and 14 to 22 m in depth with a north to south orientation. This passage contained the FA, which was situated on the north-west aspect of the passage and covered an area of ~ 3,500 m^2^ (Fig 1C). Habitats within the FA included a reef crest and reef slope, mainly dominated by tabular and digitate hard corals. The reef fronts, on both the outer and inner side of the passage also included some groove and spur morphology and both slopes terminated at ~ 20 m depth into a sand-rubble substratum dotted by isolated coral patches (Fig 1B). On 3 February 2011, a large tropical cyclone (“Yasi”, Category 5 strength) destroyed 95% of the tabular corals at the FA.

### Environmental variables

Outer reef fronts and passages experience changing environmental conditions on a daily and seasonal scale [33]. The information on several environmental variables was collected between February 2006 and November 2012. These variables included tidal state, moon phase and surface seawater temperature (SST) (for data source information see S1 Table). Two of the variables, water current direction and moon phase, require further explanation. The Moore Reef passage was similar to other reef passages such as the Ribbon Reefs in the northern GBR [34] and the Marshall Islands in the central Pacific [3], in that it experiences strong alternating currents generated by tides. At the study area, the flood tide causes water to flow onto the reef and lagoonwards, while the ebbing tide forces water to flow out through the passage to the Coral Sea (Fig 1C). Moon phase has been represented as a fraction of luminosity, which is a quantitative way of describing the moon’s phase [35]. The measurement ranges from zero to one. Zero described the new moon phase, while 1 described the full moon phase and the first and last quarters were represented by 0.5. First and last quarter can be distinguished by noting whether the fraction illuminated was increasing or decreasing. First quarter occurred when the fraction illuminated was increasing (moon waxing; in evening sky) and last quarter occurred when the fraction illuminated was decreasing (moon waning; in morning sky) [35].

### Species richness and abundance estimates

A pilot study was conducted at the study site from February 2005 to January 2006, during which time a species list of larger fish (>20 cm total length) that occur at the site was compiled (S2 Table). A subset of 10 common species from 5 families was selected for this quantitatively descriptive study because they formed relatively high density, conspicuous aggregations (Table 1). This selected suite of large coral reef fishes displayed a variety of foraging and reproductive modes. These included pelagic predators (Carangidae), predators on reef-associated fishes (Lutjanidae), egg predators (*Macolor niger*) and planktivorous species (Acanthuridae) to represent fish exploiting food characteristics of the outer reef fronts. Species representing predators on benthic invertebrates (Lethrinidae, Haemulidae and some Carangidae) were selected to potentially represent fish exploiting the outflowing currents of a reef pass to reproduce.

**Table 1:** The 10 species of coral reef fish surveyed between February 2006 and November 2012 at the fish aggregation site, Moore Reef, classified into 5 broad trophic groups.

Trophic group	Family	Species	Reference source
Predators of small fish	Carangidae	*Caranx sexfasciatus*	[36–39]
	Lutjanidae	*Lutjanus bohar*	[39]
Predators of benthic invertebrates	Carangidae	*Trachinotus blochii*	[40]
	Haemulidae	*Plectorhinchus lineatus*	[40]
	Lethrinidae	*Lethrinus nebulosus*	[39, 41]
	Lethrinidae	*Lethrinus olivaceus*	[41] (also has consuming fish and cephalopods)
	Lethrinidae	*Monotaxis grandoculis*	[36, 41, 42]
Planktivores	Acanthuridae	*Naso annulatus*	[43]
Egg predators	Lutjanidae	*Macolor niger*	[20, 44] (S1 Video)
Omnivores	Acanthuridae	*Acanthurus dussumieri*	Sand grazers [45], Detritivore [46], grazing herbivore [47], planktivore and faecal scavenger (S2 Table and S2 Video)

The abundance of the selected aggregating species was estimated 4 to 5 days a week for 6 years by the senior author. These UVC counts followed the same census path, starting at the mouth of the channel and moving north around the outer slope of the tourist pontoon reef (Fig 1C). This survey was conducted daily between 1400 h and 1500 h while on snorkel. Notes were also taken during census on the trophic or reproductive behaviour of fish present (S2 Table). Foraging behaviour was observed as the direct consumption of prey items, and with planktivores feeding behaviour was associated with the up and down movement in the water column. Courtship behaviour, such as specialised colour changes and chasing were identified from published examples [18, 48, 49].

### Caesionidae abundance

Caesionidae represents a large component of outer slope reef fish communities on mid-shelf reefs in the GBR [50]. These fish are small, mobile, can form dense aggregations, feed on plankton and have been considered too difficult to count accurately in UVC studies [30] and therefore were omitted from the original monitoring programme. Three of the five species of Caesionidae were known to form foraging aggregations at the fish aggregation site (S2 Table). To assess whether there were temporal changes in the abundance of these planktivores at the FA, a GoPro video camera was fixed to a star picket in 9m depth of water in approximately the centre of the FA. During October 2013 and February 2014, two days in each month were selected for videotaping. The two days in each month coincided with a flood and ebb tide phase, and on each day the camera recorded 4 hours of video footage. A 40-minute video from each four-hour video was selected that represented the middle of the tide phase. A random number generator was used to select 10 screen shots from each 40-minute video. The selected screen shots were used with the software Event Measure [51] to accurately count the abundance of caesionids present at a distance of up to 6m from the camera.

### Statistical analyses

The data comprised 1,357 UVC counts at the FA between 1 February 2006 and 29 of November 2012. There were 402,370 individual fish from 10 species in this dataset. Date, month and moon phase were available for all counts, and SST was measured for all but 21 samples. We performed univariate and multivariate analyses on untransformed fish counts using two types of regression trees. Regression trees can be summarised in ways that give powerful ecological insight by representing complex information in a visual format that can be easily interpreted [52, 53].

The univariate responses of species richness and raw fish counts of the 10 selected species were modelled using aggregated boosted regression trees (ABT) to summarise the relative influence of major predictors and to interpret interactions [54–56]. The ABT models included the main effects and up to three-way interactions amongst the full suite of five explanatory covariates (tide, moon phase, month, decimal date, and SST), all predictors and up to third-order interactions. No monotonic constraints were applied to the functional form of selected individual predictors. Five methods were used to interpret and compare the models as per Fabricius and De’ath [56]. The best predictive models were determined by comparing the “prediction error” (PE) expressed as a percentage of models with varying levels of interactions.

Multivariate prediction and regression trees (MRT) were used to model the abundance of all 10 fish species in response to the most influential explanatory covariates identified in the univariate models. The overall fit of the model was the “relative error” (RE), or fraction of variance not explained by the tree [52]. The RE over-estimates the performance of the tree when predicting for new data, so predictive accuracy was estimated from five-fold cross validation (the “cross-validated relative error”; CVRE). The most parsimonious models selected were the ones that simultaneously minimise RE and CVRE [52, 53]. The performance of the final model was assessed by comparing the best MRT with an unconstrained clustering of the same distance matrix with the same numbers of terminal nodes (clusters). If the unconstrained cluster analysis accounts for substantially more of the species variation than an MRT analysis, it was likely that additional, unmeasured covariates were responsible for the difference in explained species variation [52].

Each node of the tree can be defined by the multivariate mean of its samples, the predictors that define it, the number of samples that were grouped there, and by Dufrêne-Legendre species indicators (DLI). For a given species and a given tree node, the DLI was defined as the product of the mean species abundance occurring in the group divided by the sum of the mean abundances in all other groups (“specificity” A), times the proportion of sites within the group where the species occurs (“fidelity” B), multiplied by 100 [57–59]. Each species can be associated with the tree node (assemblage) where its maximum DLI value occurred. The index distinguishes between ubiquitous species that dominate many nodes in absolute abundance and species that occur consistently within single nodes, but have a low abundance [52]. Finally, the point-biserial correlation coefficient was used for determining the ecological preferences of species among the set of alternative tree nodes or node combinations. This was a generalisation of the Pearson's phi coefficient [60]. All analyses used the R open source libraries vegan, abt, mvpart, mvpartWRAP and indicspecies [61].

## Results

### Abundance

**Fig 2:**
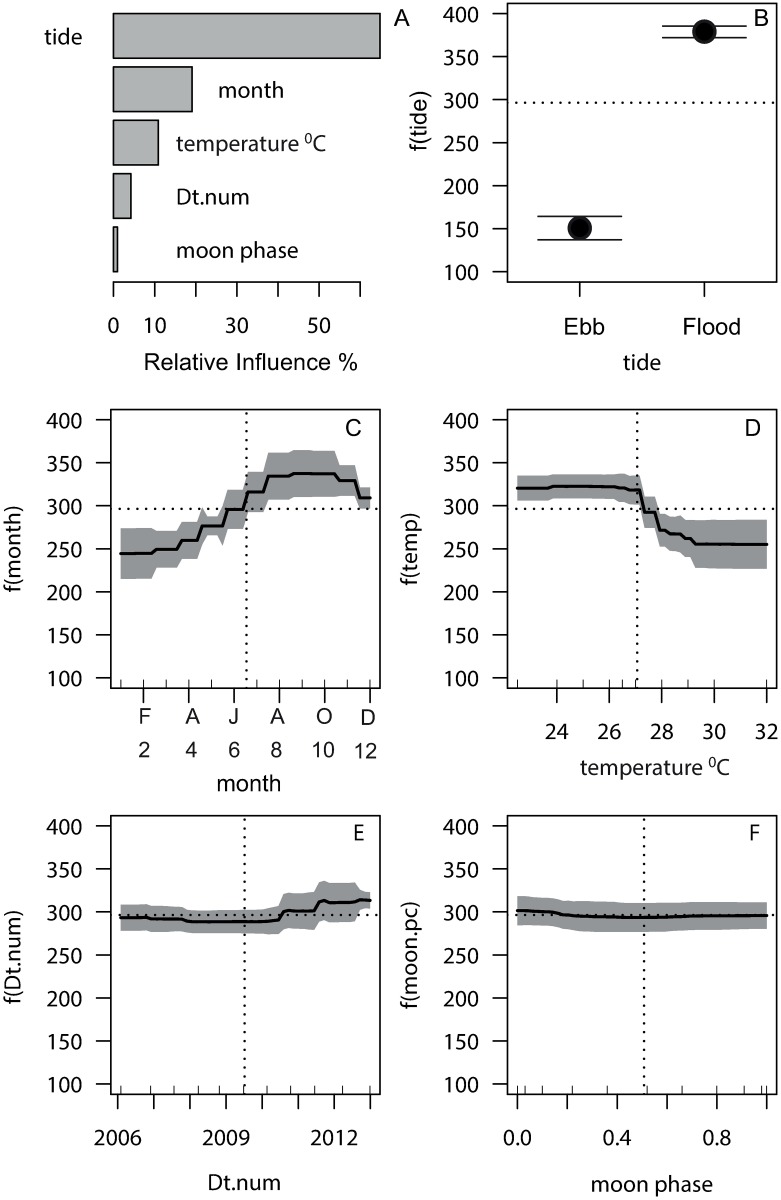
Partial dependency plots for the univariate response of pooled fish abundance from the best aggregated boosted regression tree model. Relative influences (A) of pooled fish abundance (10 selected species) in underwater visual census samples at Moore Reef fish aggregation. Partial dependency plots of fish abundance on the tidal direction (B), month (C), surface seawater temperature (D), decimal date (Dt.num) (E) and moon phase (F), show the response of abundance as a function f () of each predictor, with the influence of all other predictors held to a constant. Shading around the response lines is 2 standard errors. Horizontal dotted lines show the mean fish abundance across all counts. Vertical dotted lines show the mean value for each predictor. Rugs on the x axes show the spread of sampling in ten-percentiles within the range of each predictor.

Abundance patterns of the 10 species varied over the six-year sampling period, primarily in association with five environmental variables “tide”, “month”, “SST”, “date”, and “moon phase”. Partitioning of the five environmental variables demonstrated that tide was the dominant influence (64.8%), followed by month (19%) and SST (11%). Date, representative of among-year variation, and moon phase (frequently associated with reproductive periodicity), showed a minor influence (4% and 0.9%), respectively (Table 2 and Fig 2A). Permutation tests showed the effects of dropping these minor influences had little effect on PE, but omitting tide caused a 1.5-fold increase in PE in relation to the full model (Table 2). Partial dependency (effects plots) showed that UVC counts made at the aggregation site on flood tides had 2.3 times as many fish as counts made on ebb tides (Fig 2B).

**Table 2:** Relative influences (%) of environmental variables on total fish abundance and species richness (10 selected species) in underwater visual census counts at the Moore Reef fish aggregation site.

	Total Fish Abundance			Species Richness		
	Influence	PE%	P	Influence	PE%	P
**abt full model**		44.8			48.29	
**Tide**	64.82	150.7	<0.001	67.474	136.65	<0.0001
**Month**	18.92	27.50	<0.0001	11.098	12.69	<0.0001
**SST °C**	11.08	14.89	<0.0001	13.278	14.45	<0.0001
**Decimal Date**	4.23	4.01	<0.0001	6.96	6.81	<0.0001
**Moon phase**	0.94	0.50	<0.0001	1.1889	0.39	0.025

The top row shows the prediction error (PE%) of the best aggregated boosted regression tree models. Subsequent rows show the results of permutation tests (n = 1000 permutations) and the increase in PE% by dropping each variable, relative to the full model.

**Fig 3:**
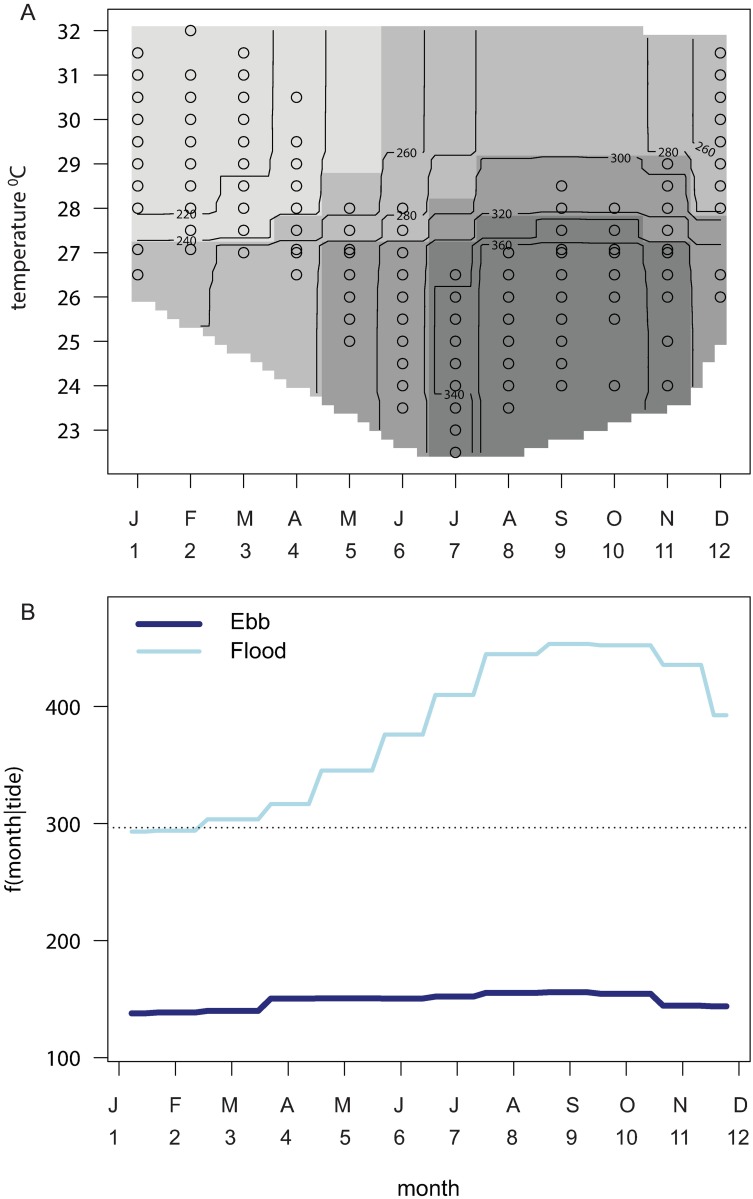
Partial interaction plots from the best aggregated boosted regression tree model for pooled fish abundance. Partial interaction plots for the response of pooled fish abundance (10 selected species) to different seawater temperatures at different months (A) and as a function f () of months given tidal direction (B). The contours of fish abundance in (A) show the spread of temperatures sampled in each month as ten-percentiles (open circles) and the increasing grey scale represents increasing fish abundance.

Fish abundance patterns of the 10 target species at the fish aggregation site were predictable. Models of main effects with 3-way interactions had a PE (44.8%) lower than that observed with models including 2-way interactions (47.6%), equating to an R^2^ of 55.2% in explaining variance in total fish abundance. There was evidence of a recurring pattern of higher fish abundance in the second half of the year (the austral spring/summer) (Fig 2C). This was heightened when SST’s were below the long-term mean (Fig 2D), indicating the influence of among-year variation in temperature. There was a trend in increasing fish abundance post-mid 2011 (Fig 2E), but lunar periodicity had no influence on fish abundance (Fig 2F). Partial interaction plots showed that UVC samples from cooler seawater temperatures in the period August-November had higher than average fish abundance (Fig 3A). Flooding tides had higher abundance than ebbing tides in all months, but that difference was greatest in the second half of the year (Fig 3B).

### Species richness

**Fig 4:**
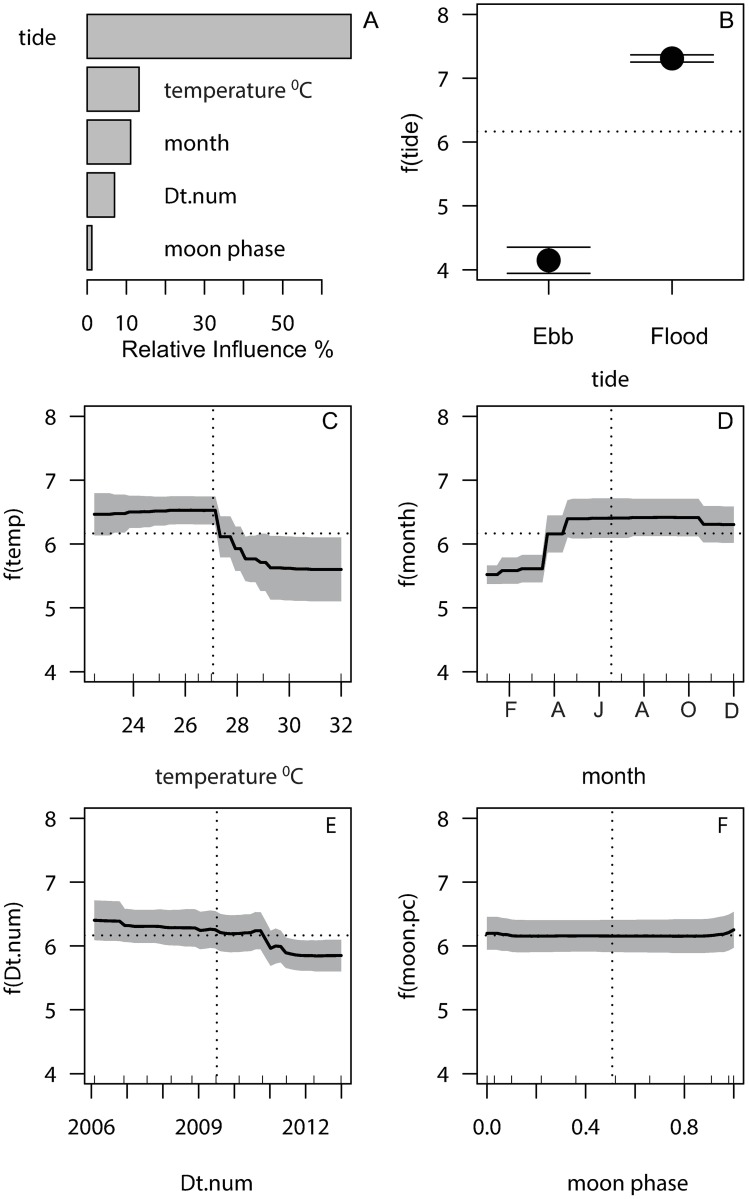
Partial dependency plots for the univariate response of species richness from the best aggregated boosted regression tree model. Relative influences (A) of species richness in underwater visual census samples at Moore Reef fish aggregation site. Partial dependency plots of species richness (10 selected species) on the tidal direction (B), surface seawater temperature (C), month (D), decimal date (Dt.num) (E) and moon phase (F), show the relationship of richness as a function f () of each predictor, with the influence of all other predictors held to a constant. Shading around the response lines are 2 standard errors. Horizontal dotted lines show the mean richness across all counts. Vertical dotted lines show the mean value for each predictor. Rugs on the x axes show the spread of sampling in ten-percentiles within the range of each predictor.

The increase in total fish abundance at the fish aggregation site was driven by an increase in species richness. However, the species richness model of the 10 selected species was different to the abundance model in the order of dominant variables influencing the observed patterns. SST (13.3%), not month (11%), was the second dominant variable influencing the number of species at the fish aggregation site (Table 2 and Fig 4A). Tide had the highest influence on species richness (67.5%), with decimal date (7%), and moon phase (1.2%) being the least influential (Table 2 and Fig 4A). Partial dependency (effects plots) showed that UVC counts made at the aggregation site on flood tides had about 1.75 times as many species counts made on ebb tides (Fig 4B). Species richness was higher in cooler months (Fig 4C) and notably when temperatures were below the long-term mean (Fig 4D). This was indicative of among-year variation in seasonal temperatures, and also a yearly effect was noted in that species richness decreased under the mean from 2011 (Fig 4E). Overall, lunar periodicity had no influence on species richness. (Fig 4F).

**Fig 5:**
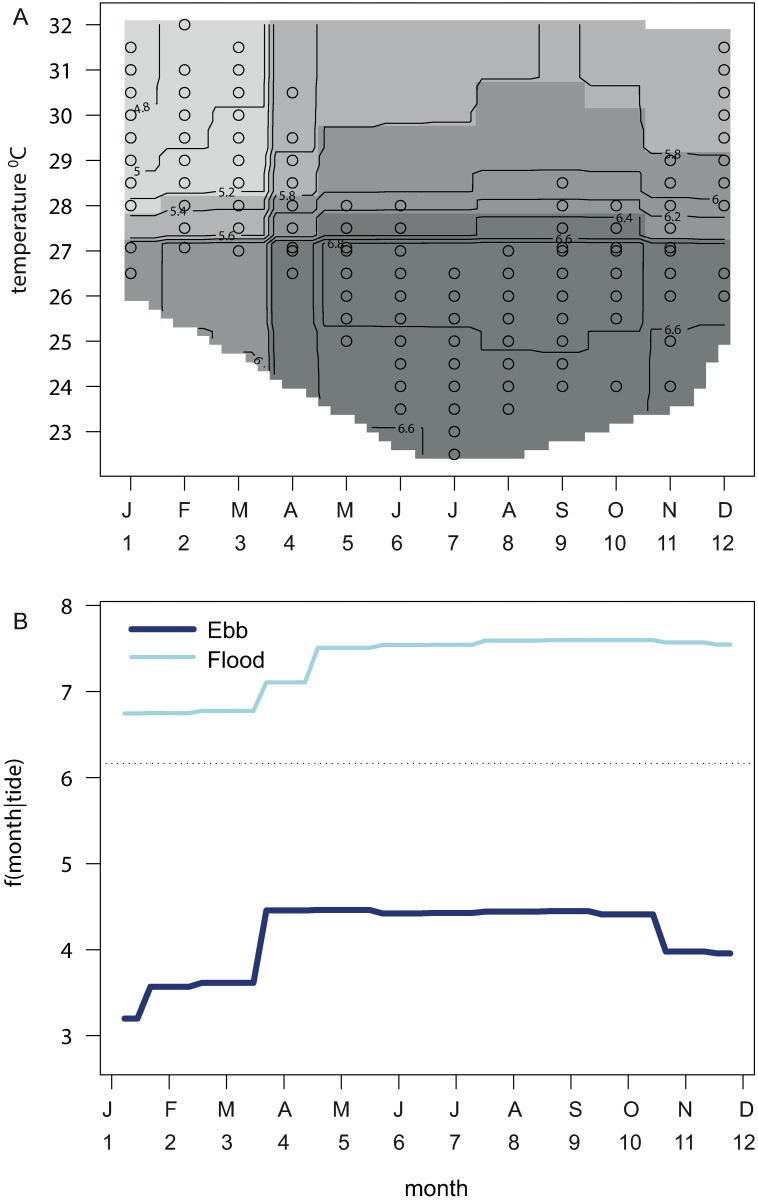
Partial interaction plots from the best aggregated boosted regression tree model for species richness. Partial interaction plots for the response of species richness (10 selected species) to different surface seawater temperatures at different months (A) and as a function f () of months given tidal direction (B). The contour plot in (A) shows the spread of temperatures sampled in each month as ten-percentiles (open circles) and increasing grey scale with increasing species richness.

The best model, with 3-way interactions, had a relative PE of 48%, equating to an R^2^ of 52% in explanation of the variance in species richness. Models of main effects (PE = 55.7%) and 2-way interactions (PE = 50.7%) had higher prediction errors. Permutation tests showed that the effects of dropping moon phase from the model had no significant effect on PE, but omitting tide caused a 1.3-fold increase in PE in relation to the full model (Table 2). The error in the model without tidal state was 1.14 times the overall variance in species richness. The interactions among the covariates of SST, month and tide were interpreted from partial interaction plots (Fig 5). Species richness was higher than average with cooler SST’s in the period May-October (Fig 5A), but higher richness on flooding tides in all months (Fig 5B).

### Assemblage structure

**Fig 6:**
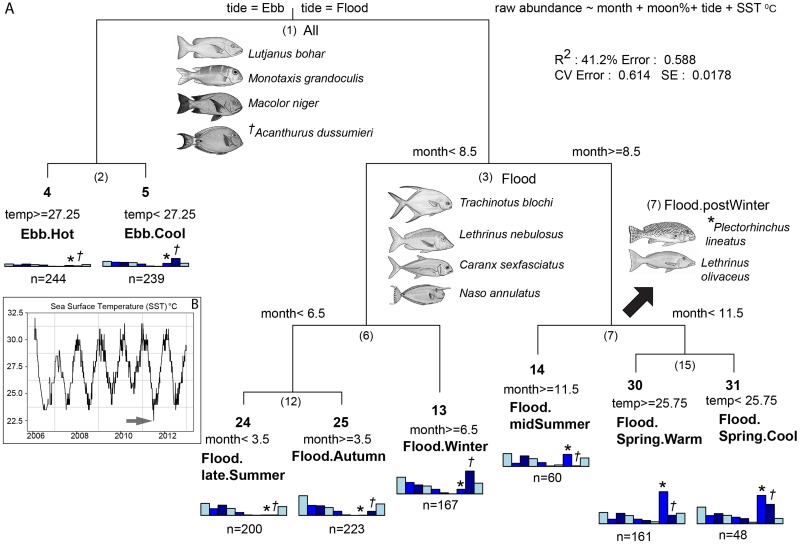
Multivariate regression tree for 10 species of fish from Moore Reef fish aggregation site. The best tree structure from a multivariate regression tree analysis of the counts of 10 species predicted by month, surface seawater temperature, tidal direction, and moon phase (A). Histograms on the terminal nodes (“leaves”) show the abundance of each species and the number of underwater visual census samples (n). The bars on the histograms, from left to right represent 1. *Trachinotus blochii*, 2. *Caranx sexfasciatus*, 3. *Lutjanus bohar*, 4. *Monotaxis grandoculis*, 5. *Macolor niger*, 6. *Lethrinus nebulosus*, 7. *Lethrinus olivaceus*, 8. *Plectorhinchus lineatus* (indicated by *), 9. *Acanthurus dussumieri* (indicated by ^ϯ^), 10. *Naso annulatus*. The node numbers are in boldface type for leaves and given in brackets for higher nodes. The DLI characterising the labelled nodes and leaves are an index of fidelity and specificity of a species to a node. (B) annual variation in surface seawater temperature, with the arrow marking the lowest recorded temperature during the 6-year study period.

Analysis of the multi-species abundance data produced recognisable assemblages of reef fishes. These assemblages displayed fidelity and predictability with respect to the five environmental variables that were incorporated into MRT. The best performing MRT split the data into eight assemblages on tide, month and SST when moon phase was included (Fig 6A). The overwhelming environmental influence on fish abundance patterns at the fish aggregation site was tide. Whether individual fish species occurred throughout the year or were seasonally represented, all 10 species, regardless of their trophic classification (Table 1), were more abundant on the flood side of the regression tree. The left hand branches were the counts made on ebb tides, with low abundance of all species (Fig 6A). The first split of the MRT explained a third of the tree variance, 14.4% of the 41.2% (Table 3).

**Table 3:** Summary of the multivariate regression tree (Fig 6).

Splits Species	1 (Tide = Ebb)	2 (Temp = 27.25)	3 (Month = 8.5)	6 (Month = 6.5)	12 (Month = 3.5)	7 (Month = 11.5)	15 (Temp = 25.75)	Tree %variance	Species %variance
*Trachinotus blochii*	4.8	0.09	0.29	0	0.72	0	0.12	6.04	16.2
*Caranx sexfasciatus*	1.01	0.11	0.43	0.12	0.13	0.03	0.06	1.87	6.79
*Lutjanus bohar*	1.65	0.04	0.01	0.01	0	0	0	1.72	3.71
*Monotaxis grandoculis*	0.93	0.06	0.01	0	0.01	0	0	1.01	2.25
*Macolor niger*	0.21	0.01	0.02	0.01	0.02	0.01	0.02	0.3	0.95
*Lethrinus nebulosus*	0.06	0	0.11	0	0	0.06	0.01	0.25	1.53
*Lethrinus olivaceus*	0.01	0	0.06	0	0	0	0	0.08	0.3
*Plectorhinchus lineatus*	2.53	0.06	13.82	0.25	0	1.99	0.06	18.71	28.78
*Acanthurus dussumieri*	0.67	0.77	0	5.78	0.16	0.58	0.51	8.47	15.97
*Naso annulatus*	2.48	0.02	0.03	0	0.09	0	0.12	2.75	23.52
Split % variance	14.35	1.17	14.79	6.19	1.14	2.66	0.9	41.2	100

The overall amount of variation in species abundance in 1,357 underwater visual census samples (Species % variance), the species-specific variation explained by the MRT (Tree %variance), the node number and threshold values for the splits (Splits), and the individual and overall variation in species abundance explained by each split (Split % variance).

The MRT model illustrated seasonal periodicities in fish abundance by producing distinct seasonal assemblages on the flood side of the tree categorised by month with the spring assemblage further divided into warm and cool, split at the threshold value of 25.7°C (Fig 6A). Monthly temperature ranges varied (Figs 3A and 5A), which was reflective of inter-annual variation in temperatures, with the lowest temperature recorded in 2011 and the highest in 2006 (Fig 6B). Although no single species was an indicator for any leaf of the tree, all indicators occurred higher in the tree at combinations of nodes and branches (Fig 6A and Table 4). This apparent discrepancy between the seasonality of abundance, but the lack of characteristic indicators for terminal seasonal leaves, may be due to the ambitious nature of the MRT. For example, *Acanthurus dussumieri* was a ubiquitous indicator at the root node, but this species showed clear peaks in abundance when seawater temperatures were cooler, on both the flood and ebb side of the tree (Fig 6A). This was supported by the relatively high point biserial coefficient of association of 0.69 with tree leaves 13 (“Flood.Winter”) and 31 (“Flood.Spring.Cool”) (Table 5) and the lack of this species from the summer assemblages (Fig 6A).

**Table 4:** The Dufrene-Legendre indicator value for each of the 10 selected species at the Moore Reef fish aggregation site.

Splits (Node)	Species	A	B	DLI	p
All (1)	*Lutjanus bohar*	0.97	0.92	0.89	0.001
All (1)	*Monotaxis grandoculis*	0.97	0.91	0.88	0.001
All (1)	*Macolor niger*	0.96	0.89	0.85	0.001
All (1)	*Acanthurus dussumieri*	0.98	0.85	0.83	0.001
Flood (3)	*Trachinotus blochii*	0.92	0.91	0.84	0.001
Flood (3)	*Lethrinus nebulosus*	0.96	0.85	0.81	0.001
Flood (3)	*Caranx sexfasciatus*	0.89	0.82	0.73	0.001
Flood (3)	*Naso annulatus*	0.92	0.6	0.55	0.001
Flood.post.Winter (7)	*Plectorhinchus lineatus*	0.89	0.93	0.83	0.001
Flood.post.Winter (7)	*Lethrinus olivaceus*	0.96	0.43	0.41	0.001

The DLI is the product of “specificity” (A) and “fidelity” (B) and the nodes for which each species had a maximum in DLI are shown with probability values p.

**Table 5:** The point-biserial correlation coefficient of association (phi) between species and tree node.

Splits (Node)	Tree Leaves	Species	phi	p
Flood (3)	13+14+24+25+30+31	*Lutjanus bohar*	0.63	0.001
Flood (3)	13+14+24+25+30+31	*Monotaxis grandoculis*	0.60	0.001
Flood (3)	13+14+24+25+30+31	*Trachinotus blochii*	0.48	0.001
Flood (3)	13+14+25+30+31	*Macolor niger*	0.45	0.001
Flood (3)	13+14+24+25+30	*Naso annulatus*	0.26	0.001
Flood (3)	13+24+25+31	*Caranx sexfasciatus*	0.46	0.001
Flood (3)	14+30+31	*Lethrinus olivaceus*	0.45	0.001
Flood.Spring (15)	30+31	*Plectorhinchus lineatus*	0.73	0.001
Flood.Spring (15)	30+31	*Lethrinus nebulosus*	0.39	0.001
Flood (3)	13+31	*Acanthurus dussumieri*	0.69	0.001

Each node and terminal leaf of the tree was defined by the predictors, the number of samples that were grouped there, and by DLI in Fig 6A. Two species *Lethrinus olivaceus* and *Plectorhinchus lineatus*, both predators of benthic invertebrates, also showed predictable seasonal patterns of occurrence. Both *L*. *olivaceus* and *P*. *lineatus* had the highest DLI with the combination of leaves 14, 30 and 31 on the right hand branches in the MRT with node 7 (“Flood.post.Winter”). The “specificity” (A) of 0.96 for *L*. *olivaceus* at this node represented a 96% probability that any particular UVC count recording *L*. *olivaceus* would occur in this node. However, the “fidelity” of 0.43 showed that *L*. *olivaceus* occurred in only 43% of UVC samples in this node. In contrast, *P*. *lineatus* had both high specificity and a high fidelity (93% of samples in this node) (Table 4). The point-biserial coefficient of association between each species and nodes showed a relatively high association (0.73) between the abundance of *P*. *lineatus* and the node 15 “Flood.Spring” and leaves 30 and 31 of the MRT, regardless of differences in spring water temperatures (Table 5 and Fig 6A).

The fish assemblages of all ten species on diel or seasonal time-scales were predictable. The MRT had a prediction error of 61% and a relative error of 58.8% equating to an explanation of 41.2% of the multivariate distance variation. This means that the model was able to predict 4 out of 10 times, the occurrence of all 10 species at any temporal scale, ranging from hours to years. A comparison of this MRT partitioning with an unconstrained clustering of the species for all tree sizes from 1 to 7 splits (8 leaves) showed a consistently lower performance of the MRT (by approximately 50%) in explaining variation in the assemblage structure. An unconstrained clustering with 8 groups had an error of 29.5%, nearly half of that for the MRT with 8 leaves (58.8%). This implied there were other environmental covariates, and perhaps some sampling biases, that had not been included as predictors in the MRT model. Although, there was not a close match between the species contributing most to the MRT and the overall variation in abundance. For example, *P*. *lineatus* comprised 28.8% of the variation in UVC counts, and contributed nearly half (18.7%) of the distance variation explained by the MRT (R^2^ = 41.2%), yet the same ratio for *N*. *annulatus* was 23.5%: 2.8%, which translates to explaining only 7% of the distance variation explained by the MRT (Table 3).

### Caesionidae abundance

**Fig 7:**
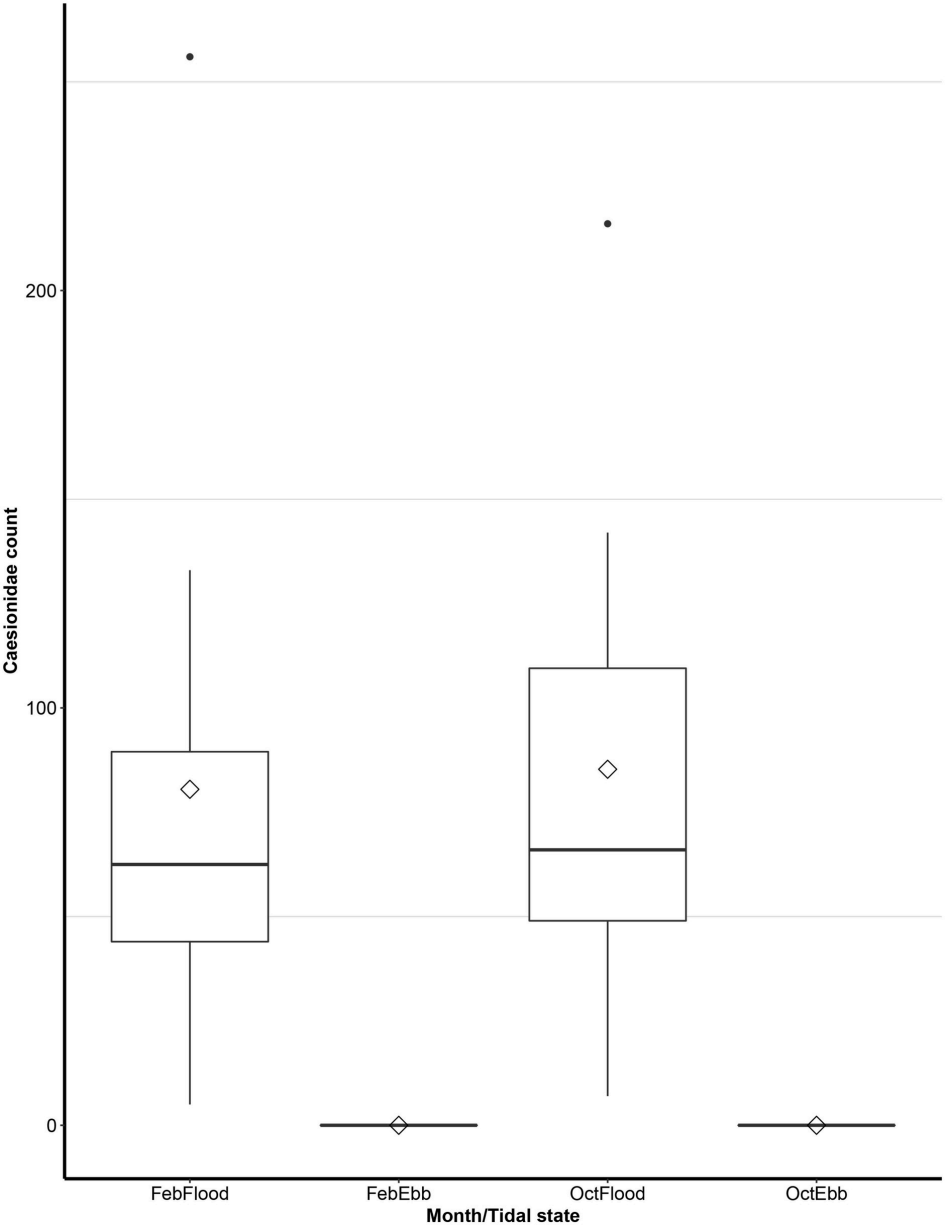
Caesionidae abundance at Moore Reef fish aggregation site. Box plot of Caesionidae abundance on flood and ebb tides for the months of February and October. The black line in box represents the median and whiskers indicate the variability outside the upper and lower quartiles. The diamond represents the mean of the samples and the black circles at the end of boxplot represent outliers.

The small planktivorous caesionids were significantly more abundant at the fish aggregation site through the flood rather than ebb tides, regardless of the two months sampled (Fig 7). No members of the Caesionidae family were recorded on ebb tides and both flood tide groups sampled produced outliers, indicating high abundance of caesionids during flood tide (Fig 7).

## Discussion

This long-term monitoring study is one of the few studies to analyse the temporal periodicities of fish aggregations on time scales that range from hours to years. The structure of fish assemblages showed high levels of periodicity, with events strongly linked to the local hydrodynamics. The primary influence was the short-term semidiurnal tidal pattern characteristic of this region of the GBR [34]. Both ebb and flood tides were expected to influence the formation of reef fish aggregations. However, the analysis showed that the flooding tide was the dominant environmental factor associated with fish aggregating. Flood tides correlated strongly with several species forming foraging aggregations. Seasonal patterns in abundance and species richness of the selected species, not normally associated with outer reef habitats for foraging, may suggest a reproductive signature. Surprisingly, moon phase often associated with fish forming spawning aggregations [1, 19, 28] contributed very little to the overall structure of the fish assemblages. The results suggest a strong interaction between fish abundance and cooler SST reflective of seasonal preference of some aggregating species and inter-annual variation in total fish abundance of all 10 species. Species richness and abundance of all 10 species were highest throughout late winter and spring (Austral calendar). The study also suggests strong trophic links between aggregating functional groups, which included small planktivores with piscivores, and coprophagous fish with piscivores and invertivores.

Flood tides correlated strongly with the formation of fish aggregations at the site, wherein fish probably capitalise on the ingress of oceanic plankton with the incoming tide. Outer reef slopes vary in configuration due to the presence of grooves, spurs and passages and these morphological features can influence the oceanic forcing of water onto the hard reef [33]. Local topography, such as narrow passes between reefs, may entrain deep flowing water rich in zooplankton near the reef crest. Flood tides in a reef passage typically cause upwelling of water in front of the reef, which are then carried through the passage by tidal jets [8]. The surface waters that enter the passage are drawn radially from a semi-circle of the ocean that was centered at the entrance of the passage and flow separation occurs at the two points on the upstream side of the passage [21]. The FA occurs at one of these separation points (Fig 1C), and water movement in this location on flood tides has been recorded to be slower compared to the passage and further along the outer reef slope (EE. Fisher, unpubl data). Like spur and groove morphology [30], separation points may entrain planktonic material in the immediate area on flood tides for longer periods. The repeated occurrence of small and large planktivorous fish at the FA on flood tides provides some indirect evidence that there are regular periodicities in the importation of allochthonous plankton on flood tides.

In support of our predictions, the inputs of small plankton and nekton with flood tides supported the continual presence of planktivores and predators of small fish. The small planktivores from the family Caesionidae were more abundant at the FA on flood tides than ebb tides. Likewise, short-term temporal patterns in abundance were shown for the two predators of small fish, *Lutjanus bohar* and *Caranx sexfasciatus*. Both predators were consistent members of all flood assemblages and known to forage on small oceanic and reef associated fishes at the FA (S2 Table). The large planktivore *N*. *annulatus*, a consumer of larger planktonic items including gelatinous plankton [43], was also a consistent member of all flood assemblages at the FA. However, the analysis only explained 7% of the variation in the occurrence of *N*. *annulatus* in relation to the environmental covariates examined. This implies other variables that have not been accounted for, such as the unpredictable boom-bust cycles of gelatinous zooplankters [6] and possibly other large-scale hydrodynamic processes [10, 62], may influence the abundance of preferable planktonic prey for *N*. *annulatus*. Flood tides at the FA suggest coral reef fish potentially form foraging aggregations derived from oceanic resources.

The higher fish abundance and species richness of the 10 selected species in spring were a general feature of the six-year monitoring program and are suggestive of some species forming aggregations for reproductive purposes. The two species classed as predators of benthic invertebrates (*L*. *olivaceus* and *P*. *lineatus*) peaked in abundance during spring flood tides. Neither species was observed spawning on flood tides, although individuals of both species were observed to have swollen abdomens and appeared gravid (S2 Table), which has been considered as indirect evidence of imminent reproductive activity [63]. The latter species occurred in consistently large numbers during the spring each year of the monitoring period. There is indirect evidence that some members of the Haemulidae family form spawning aggregations in the west Indian Ocean [64] and the west Pacific Ocean [65]. While there is a lack of published information on the reproductive behaviour of any member of the Haemulidae, histological information from a western Atlantic Haemulid representative shows that, *Haemulon plumieri* collected from higher latitudes (> 17°) has a distinct spawning season [66, 67].

The species *L*. *olivaceus* was also seasonal in appearance at the FA, with peaks in abundance during spring flood tides, suggestive that spawning may be the reason for their aggregation. Histological evidence from other studies indicates that some species of the lethrinids on the GBR have a distinct spawning season in the cooler periods of the year [68–70], though details on mating systems of lethrinids on the GBR are lacking. In other locations, several species of the lethrinids are suggested to form spawning aggregations [64, 65, 71], and histological evidence and acoustic telemetry suggested that *Lethrinus harak* made nightly spawning migrations coinciding with lunar and tidal cues in the Pacific island of Guam [72]. Nocturnal spawning has possible evolutionary advantages due to reduced egg predation [1]. However, it is difficult to observe, possibly explaining why there is only one documented account of lethrinids aggregating to spawn [73]. Spawning at dusk or night has also been suggested for some members of the family Haemulidae from observations of oocyte development [74].

The indirect evidence of repeatable seasonal aggregations of *P*. *lineatus* and *L*. *olivaceus* at the FA are suggestive of fish forming spawning aggregations. Neither species normally forages on upper outer reef slopes [40], and observations made during the present study found that both aggregations contained fish with swollen abdomens (S2 Table). The question is why both species are more abundant on flood rather than ebb tides. We hypothesise the FA may act as a staging or courtship arena, increasing the probability of social interaction and mate selection [75], and the tidal cue of flooding may synchronise these behaviours [76]. Lunar periodicity has been shown to synchronise spawning behaviour in some lethrinid species [72, 73], although this was not a finding in the present study.

At the FA, SST was found to influence the abundance and species richness of fishes. Abundance and richness of the 10 selected species were highest when SST was below the long-term mean of 27°C. For abundance data, a yearly effect was detected with the highest abundance of all 10 species being recorded in the spring months of 2011 when the inter-annual SST was lowest for the monitoring period. The omnivore, *A*. *dussumieri*, showed species-specific thermal preferences and was highly abundant when water temperature was below 25.7°C. At this time, the species was seen to consume protein-rich faeces of piscivores and invertivores [27] (S2 Table and S2 Video). The increase in abundance of *A*. *dussumieri* correlated with an increase in species richness of higher trophic groups and was suggestive of a tight interaction between aggregating species and fishes utilising resource pulses created by aggregating fishes. This study suggests coral reef fish aggregations could be influenced by large-scale meteorological events, such as El Niño, by producing warmer SST and as well as by anthropogenic climate change [17].

Tropical cyclones may also influence coral reef fish aggregations. At the FA, species richness showed an opposite trend to overall fish abundance with the lowest values recorded in 2011 and 2012. The trend in dropping richness may be associated with the high destruction of tabular corals at the fish aggregation site by the passage of the tropical cyclone “Yasi” in 2011. Tabular corals have been identified as important habitat for large mobile coral reef fish including the families Haemulidae, Lethrinidae and Lutjanidae and Acanthuridae (including *A*. *dussumieri* and *Naso sp*.) [77]. The reduction in this important habitat type may have influenced the occurrence and abundance of 8 of the 10 species selected in this descriptive study.

## Conclusions

Flood tides were the dominant environmental driver underlying the formation of aggregations by 10 large coral reef fishes on an outer slope adjacent to the seaward side of a passage of Moore Reef. The importance of flood tides to these aggregations emphasises the necessity to incorporate tide into the sampling design of monitoring studies to determine long-term changes in fish assemblages [78]. The study found that some species aggregated at the site on a daily basis and others were more seasonal in occurrence. The daily occurrence of small and large planktivores and piscivores were likely to be associated with fishes forming short-term foraging aggregations. In contrast, the seasonal presence of two species of benthic feeding invertivores appeared to be related to fishes forming spawning aggregations. Contrary to our predictions, flood tides were the dominant correlates of the possible trophic and reproductive aggregations. This comprehensive study, despite being limited to one site, does provide a detailed description of the temporal patterns of fish aggregations in relation to the physical environment and hypothesises the biological motive for trophically different species forming aggregations. To resolve this requires more specific information on their trophic and reproductive biology. Future work is planned in this area, but presently restricted by permits that allow large-scale collections. Research at more aggregation sites is also warranted to test the hypotheses that some fish from certain trophic groups aggregate to spawn and others to forage. Such research may also determine the generality of our finding that reef configuration and hydrodynamics are the most important physical drivers underlying coral reef fish forming aggregations.

## Supporting information

S1 Table Describes the source of environmental conditions collated with the fish composition and abundance data. The data was collected between 1 February 2006 to 29 November 2012, except the Secchi data which commenced 24 June 2007. Data collection for some variables marked with an * was collected sporadically and not continuous due to repository limitations.
(DOCX)

S2 Table Species list of Moore Reef fish aggregation site. This list 110 fish (>20cm) from 21 families (in bold) and yes (Y) or no (N) whether these species form aggregations at the Moore reef fish aggregation site (with notes on foraging and reproductive behaviour of aggregating species or if unknown).
(DOCX)

S1 Video Faecal scavenging by Acanthurus dussumieri from predators of benthic invertebrates. (MP4)

S2 Video Egg predation by Macolor niger on Acanthurus lineatus group spawning aggregations. (MP4)

S1 Data Moore Reef fish abundance and environmental variable data. (CSV)

S2 Data Caesionidae abundance data at Moore Reef fish aggregation site. (CSV)
