# Supplementary material for: Relative influence of environmental factors on the timing and occurrence of multi-species coral reef fish aggregations
Source: PLoS One. 2018 Dec 21;13(12):e0209234. doi: 10.1371/journal.pone.0209234 (PMC6303027; doi:10.1371/journal.pone.0209234)
Supplement: S1 Table — The data was collected between 1 February 2006 to 29 November 2012, except the Secchi data which commenced 24 June 2007. Data collection for some variables marked with an * was collected sporadically and not continuous due to repository limitations. (DOCX) [file pone.0209234.s001.docx]

| Environmental variable | Source | Units | Distance and direction of source from aggregation site |
| --- | --- | --- | --- |
| Tidal Flow (Describing the direction of water flowing through the passage) | Senior Author | Flooding or Ebbing | On site |
| Moon phase described as Lunar Luminosity (the fraction of the moon’s visible disc illuminated by the sun every day between 1700 and 2100 hrs) | United States Naval Observatory astronomical department http//aa.usno.many.mil/data/. | Values between 0 and 1 with 2 degrees of precision. Tables use Chamorro Standard Time (Guam, Pacific Ocean) Similar to AEST (UTC + 10:00) |  |
| Surface seawater temperature | Thermometer suspended ½ m below surface. | °C Readings taken ~ 1500 hrs | 350 m SSW |
