# Supplementary material for: Relative influence of environmental factors on the timing and occurrence of multi-species coral reef fish aggregations
Source: PLoS One. 2018 Dec 21;13(12):e0209234. doi: 10.1371/journal.pone.0209234 (PMC6303027; doi:10.1371/journal.pone.0209234)
Supplement: S2 Table — This list 110 fish (>20cm) from 21 families (in bold) and yes (Y) or no (N) whether these species form aggregations at the Moore reef fish aggregation site (with notes on foraging and reproductive behaviour of aggregating species or if unknown). (DOCX) [file pone.0209234.s002.docx]

| Species List | Aggregation | Behaviour |
| --- | --- | --- |
| **Acanthuridae**  *Acanthurus dussumieri* | Y | Foraging – coprophagy, plankton and hard substrate  Reproductive –courtship (specialised colour patterns and chasing) |
| *Acanthurus lineatus* | Y | Reproductive – group spawning, egg predation by *Macolor niger* |
| *Acanthurus mata* | Y | Foraging - plankton |
| *Acanthurus nigricans* | N |  |
| *Acanthurus nigricauda* | Y | Foraging - hard substrate |
| *Acanthurus nigrofuscus* | Y | Reproductive – group spawning, egg predation by *Macolor niger* |
| *Acanthurus xanthopterus* | Y | Reproductive - courtship (specialised colour patterns and chasing) |
| *Ctenochaetus striatus* | Y | Reproductive – group spawning, egg predation by *Macolor niger* |
| *Zebrasoma veliferum* | N |  |
| *Naso annulatus* | Y | Foraging - plankton |
| *Naso brachycentron* | N |  |
| *Naso brevirostris* | N |  |
| *Naso lituratus* | Y | Unknown |
| *Naso tuberosus* | N |  |
| *Naso unicornis* | Y | Unknown |
| *Naso vlamingii* | Y | Foraging - plankton |
| **Balistidae**  *Balistoides viridescens* | Y | Reproductive - courtship and chasing |
| *Odonus niger* | N |  |
| **Caesionidae**  *Caesio caerulaurea* | Y | Foraging - plankton and fish eggs (labrid) |
| *Caesio cuning* | Y | Foraging - plankton and fish eggs (labrid) |
| *Caesio lunaris* | N |  |
| *Caesio teres* | N |  |
| *Pterocaesio marri* | Y | Foraging - plankton and fish eggs (labrid) |
| **Carangidae**  *Alectes ciliaris* | N |  |
| *Carangoides ferdau* | N |  |
| *Carangoides fulvoguttatus* | Y | Unknown |
| *Carangoides gymnostethus* | N |  |
| *Caranx ignobilis* | Y | Unknown |
| *Caranx lugubris* | N |  |
| *Caranx melampygus* | N |  |
| *Caranx papuensis* | N |  |
| *Caranx sexfasciatus* | Y | Foraging – Clupeidae schools |
| *Elagatis bipinnulata* | N |  |
| *Megalaspis cordyla* | N |  |
| *Scomberoides tol* | N |  |
| *Trachinotus blochii* | Y | Foraging - plankton (pteropods) |
| **Chanidae**  *Chanos chanos* | Y | Unknown |
| **Diodontidae**  *Diodon hystrix* | N |  |
| *Diodon liturosu*s | N |  |
| **Epinephlidae**  *Cromileptes altivelis* | N |  |
| *Epinephelus fuscoguttatus* | N |  |
| *Epinephelus lanceolatus* | N |  |
| *Epinephelus tukula* | N |  |
| *Plectropomus areolatus* | N |  |
| *Plectropomus laevis* | N |  |
| *Plectropomus leopardus* | N |  |
| **Ephippidae**  *Platax pinnatus* | N |  |
| *Platax teira* | N |  |
| **Haemulidae**  *Diagramma pictum* | N |  |
| *Plectorhinchus chaetodonoides* | N |  |
| *Plectorhinchus diagrammus* | N |  |
| *Plectorhinchus gibbosus* | N |  |
| *Plectorhinchus lineatus* | Y | Reproductive – swollen abdomens and chasing |
| *Plectorhinchus obscurum* | N |  |
| **Kyphosidae**  *Kyphosus cinerascens* | Y | Unknown |
| *Kyphosus vaigiensis* | Y | Foraging – plankton (surface slicks) |
| **Labridae**  *Bodianus loxozonus* | N |  |
| *Bolbometopon muricatum* | Y | Foraging – day, hard substrate |
| *Cetoscarus bicolor* | N |  |
| *Cheilinus undulatus* | Y | Reproductive - lek-based and pair spawning |
| *Chlorurus microrhinos* | N |  |
| *Chlorurus bleekeri* | N |  |
| *Hipposcarus longiceps* | Y | Reproductive – initial phase male spawning in groups |
| *Scarus altipinnus* | Y | Foraging - hard substrate |
| *Scarus dimidiatus* | N |  |
| *Scarus frenatus* | N |  |
| *Scarus ghobban* | N |  |
| *Scarus oviceps* | N |  |
| *Scarus rivulatus* | Y | Foraging – hard substrate |
| *Scarus ruibroviolaceus* | N |  |
| *Scarus schlegeli* | N |  |
| *Scarus sordidus* | Y | Reproductive – pair spawning |
| *Scarus spinus* | N |  |
| **Lethrinidae**  *Gymnocranius sp* | N |  |
| *Lethrinus erythracanthus* | N |  |
| *Lethrinus nebulosus* | Y | Unknown |
| *Lethrinus olivaceus* | Y | Reproductive – swollen abdomen and chasing |
| *Lethrinus xanthochilus* | Y | Unknown |
| *Monotaxis grandoculis* | Y | Foraging – plankton (pteropods) |
| **Lutjanidae**  *Aprion virescens* | N |  |
| *Lutjanus argentimaculatus* | N |  |
| *Lutjanus bohar* | Y | Foraging - caesionids, clupeids, juvenile *Scarus sordidus* schools and plankton (pteropods) |
| *Lutjanus fulviflamma* | N |  |
| *Lutjanus fulvus* |  |  |
| *Lutjanus gibbus* | Y | Unknown |
| *Lutjanus rivulatus* | N |  |
| *Lutjanus russelli* | N |  |
| *Macolor macularis* | N |  |
| *Macolor niger* | Y | Foraging - plankton (including pteropods) and fish eggs (Acanthuridae) |
| *Symphoricthys spilurus* | Y | Unknown |
| *Symphorus nematophorous* | N |  |
| **Monocanthidae**  *Aluterus scriptus* | N |  |
| **Ostraciidae**  *Ostracion cubicus* | N |  |
| **Pomocanthidae**  *Pomocanthus semicirculatus* | N |  |
| *Pomocanthus sextriatus* | N |  |
| *Pomocanthus xanthometopon* | N |  |
| **Scombridae**  *Euthynnus affinis* | N |  |
| *Grammatorcynus bicarinatus* | N |  |
| *Scomberomorus commerson* | N |  |
| *Thunnus obesus* | N |  |
| *Thunnus tonggol* | N |  |
| **Siganidae**  *Siganus corallinus* | N |  |
| *Siganus doliatus* | N |  |
| *Siganus vulpinus* | N |  |
| **Sphyraenidae**  *Sphyraena barracuda* | N |  |
| *Sphyraena jello* | Y | Unknown |
| *Sphyraena putnamiae* | Y | Unknown |
| **Tetradontidae**  *Arothron caeruleapunctatus* | N |  |
| *Arothron mappa* | N |  |
| **Zanclidae**  *Zanculus cornutus* | Y | Unknown |
